# Triauxic growth of an oleaginous red yeast *Rhodosporidium toruloides* on waste ‘extract’ for enhanced and concomitant lipid and β-carotene production

**DOI:** 10.1186/s12934-018-1026-4

**Published:** 2018-11-19

**Authors:** Gunjan Singh, Sweta Sinha, K. K. Bandyopadhyay, Mark Lawrence, Debarati Paul

**Affiliations:** 1 Amity Institute of Biotechnology, Amity University, Sec 125, Noida, Uttar Pradesh 201313 India; 2 Basic Sciences, College of Veterinary Medicine, Mississippi State University, Mississippi State, MS 39762 USA

**Keywords:** *R. toruloides*, Oleaginous yeast, Waste ‘extract’, Triauxic, Lipid, Carotenoids

## Abstract

**Background:**

Vegetable ‘mandi’ (road-side vegetable market) waste was converted to a suitable fermentation medium for cultivation of oleaginous yeast *Rhodosporidium toruloides* by steaming under pressure. This cultivation medium derived from waste was found to be a comparatively better source of nutrients than standard culture media because it provided more than one type of usable carbon source(s) to yeast.

**Results:**

HPLC results showed that the extract contained glucose, xylose and glycerol along with other carbon sources, allowing triauxic growth pattern with preferably usage of glucose, xylose and glycerol resulting in enhanced growth, lipid and carotenoid production. Presence of saturated and unsaturated fatty acid methyl esters (FAMEs) (C_14-20_) in the lipid profile showed that the lipid may be transesterified for biodiesel production.

**Conclusion:**

Upscaling these experiments to fermenter scale for the production of lipids and biodiesel and other industrially useful products would lead to waste management along with the production of value added commodities. The technique is thus environment friendly and gives good return upon investment.
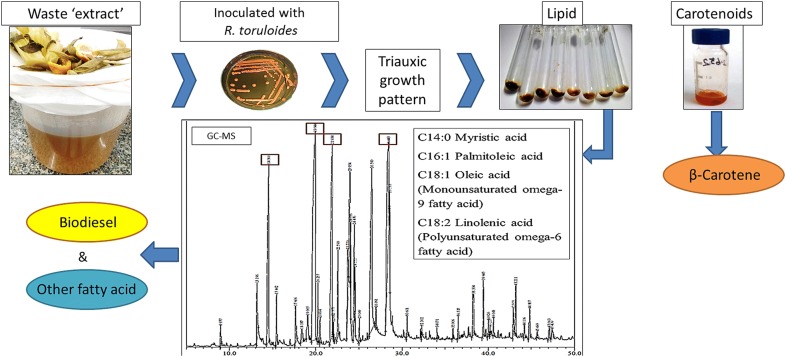

**Electronic supplementary material:**

The online version of this article (10.1186/s12934-018-1026-4) contains supplementary material, which is available to authorized users.

## Background

Rising concerns on elevated energy prices and environmental deterioration has led to a search for environment-friendly alternatives to traditional energy fuels, and in this aspect, biodiesel has proved to be one of the most promising potential substitutes. In biodiesel, the content of hydrocarbons responsible for pollution is in very low amounts. The net emission of carbon dioxide was 0% when pure biodiesel is used, as compared to 15.65% emission in a 20% blended-biodiesel sample [1–3]. Biodiesel is also less corrosive, easier to transport or store than conventional petroleum and is hence considered as a clean and renewable alternative. European Union (EU) data showed that EU accounts for 53% of the world’s biodiesel production in 2010 [4]. It has been projected by the International Energy agency that by 2050, biofuels will be able to meet at least a quarter of world’s fuel demand [5]. This implies that there is an immense boost for evolving biofuels as an alternate energy resource. Various feedstock can be used to produce biodiesel including oil extracted from plants or animals [6, 7], however, plant based or animal based oils contest for water and land, otherwise utilized for farming practices. They also compete with food which is undersupplied to some third world nations [8]. To address the above issues, microbial oil is now being considered as a feed for its low cost and environment friendly processing during biodiesel production [9]. Microbial oils exhibit several advantages over vegetable oils, due to the short doubling times exhibited by microbes and the growth is less impacted by spatial and temporal factors and readily responds to scale up as required [10].

Oleaginous yeasts, such as *R. toruloides* that accumulate lipids up to 50% of their body weight, have been reported to grow on various cheap carbon sources including industrial effluents, and are invaluable sources of triacylglycerides (TAG’s). Yeasts have advantages over algae and molds due to faster growth rate and can be easily cultivated on large scale [11]. A study by Cheirsilp et al. [12] in Southern Thailand, shows that effluents from natural rubber, palm oil and biodiesel processing industries, contain high amounts of organic load contributing to significant chemical oxygen demand (COD). Other waste material i.e. serum latex from the rubber latex concentrate industry, crude glycerol from biodiesel plant and molasses from sugarcane plant; all potentially serve as carbon-rich substrate for growing oleaginous yeast strains [12]. Palm oil mill effluent (rich in minerals and vitamins) is another waste medium, effectively used for growing oleaginous yeasts such as *Yarrowia lipolytica* [13]. In India, rich agricultural resources lead to the generation of ~ 50 MT of vegetable and fruit waste per annum, which may be considered for utilizing and growing microorganisms for biofuels [14–16]. Production of yeast-oil using agro-industrial waste [17], food waste/municipal wastewater as feedstock have been cited as ‘promising techniques’ for producing alternate energy [18, 19] and is considered as a valuable feedstock for yeast-oil.

The fatty acids mostly accumulated are palmitic acids (C16:0), stearic acid (C18:0), linoleic acid (C81:2), linolenic acid (C18:3), etc. amongst other FAMEs compounds and such combinations of long chain FAMEs are similar to vegetable oils and preferred for biodiesel production [20]. Red yeasts also produce carotenoids that are active as anti-cancer agents, as singlet oxygen foragers, as resistant reaction stimulants and furthermore as additives for makeup [21]. The application of chemical synthetic methods to prepare carotenoid compounds as food additives have been strictly regulated, leading to increased interests on finding biotechnological solutions. Several strains such as *Rhodosporidium*, *Phaffia*, *Cystofilobasidium*, etc. are known to produce carotenoids, amongst which *β*-carotene, torulene, torularhodin are the main components found in *Rhodosporidium* [22].

In this study, we have successfully cultivated *R. toruloides* using waste ‘extract’ obtained by steaming fruit peels and inedible/discarded parts of vegetables e.g. cauliflower stubs, pea pods, etc. which are generally discarded and pollute vegetable markets (mandis) in India. The lab scale biorefinery has been developed to convert waste material to useful bio-products such as biodiesel and carotenoids, with zero discharge. The FAMEs obtained from *R. toruloides* included mixtures of long chain fatty acids e.g. linoleic, oleic acid, and palmitic acid that have potentials in soap and cosmetics industry apart from being important components of biodiesel. Carotenoids extracted from *R. toruloides* showed the presence of a mixture of various components (β-carotene, torularhodin, torulene, etc.), β-carotene being the major component [23].

This bioprocess described in this study can be potentially used in future for cultivating various industrially important microbes that are capable of utilizing more than one carbon source present in the culture medium. The study also suggests an economical method for utilization of different types of waste particularly from ‘mandis’ that otherwise contaminate market places and give off foul odour by decomposing and facilitating rapid proliferation of house flies, roaches, etc.

## Materials and methods

### Microorganism and cultivation

*Rhodosporidium toruloides* (ATCC 204091) obtained from the College of Veterinary Medicine, Mississippi State University, USA was used for all experiments [24]. The yeast was grown and maintained on culture plates containing minimal media (MM): glucose 5 g/L; di-sodium hydrogen phosphate dehydrate 6 g/L; sodium chloride 5 g/L; potassium dihydrogen phosphate 3 g/L; ammonium chloride 2 g/L; magnesium sulphate 0.1 g/L; yeast extract 2 g/L; agar 1.6% (w/v); pH 5.5 [25, 26].

### Preparation of waste ‘extract’

**Fig. 1:**
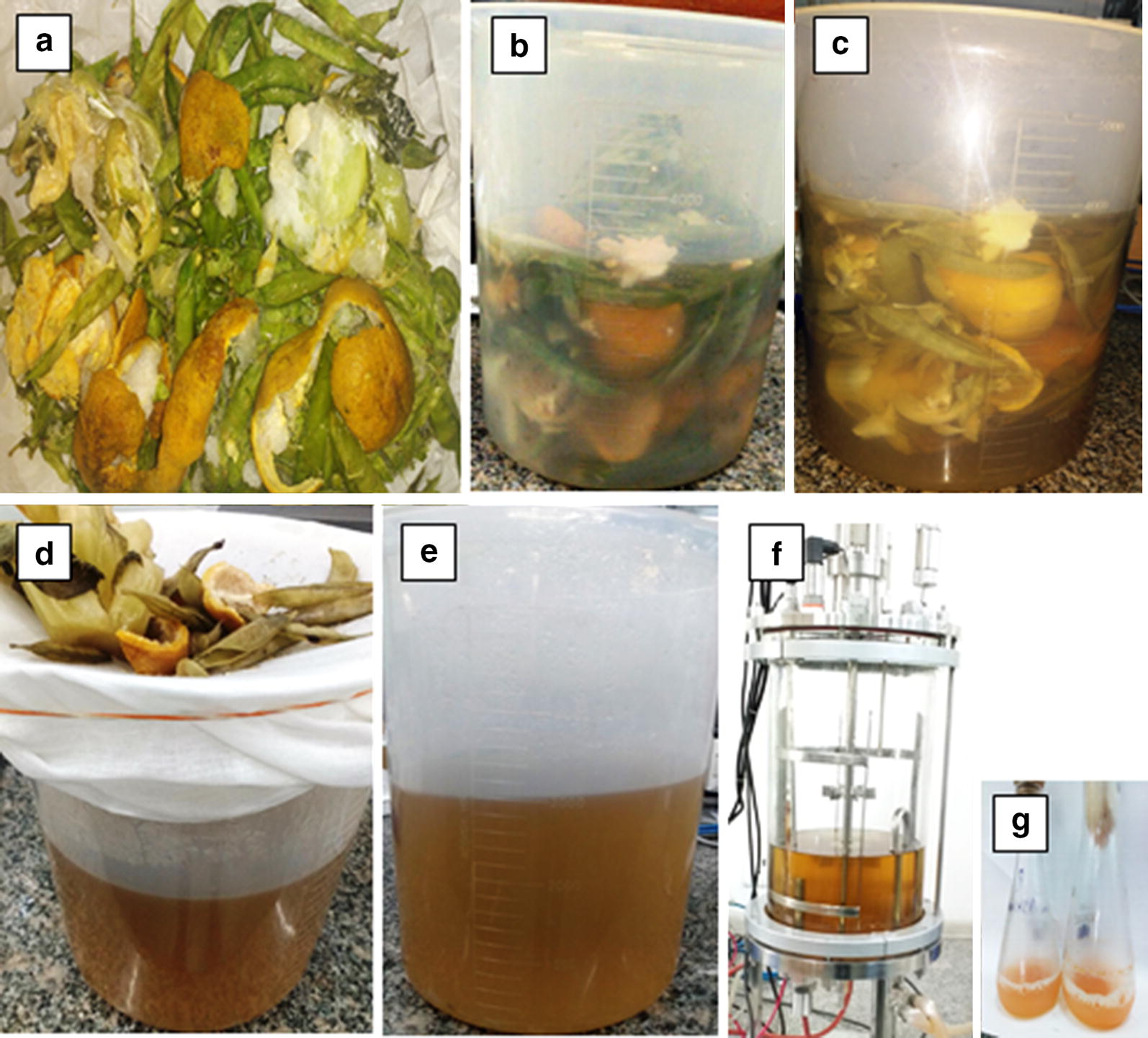
Waste ‘extract’ preparation using **a** peels and stubs of fruits and vegetables; **b** distilled water added with measured amount of waste; **c** autoclaved; **d** filtered using muslin cloth; **e** filtered waste ‘extract’ for fermentation medium; **f** waste ‘extract’ as fermentation medium and **g** yeast grown in shake flasks containing waste ‘extract’

Inedible matter (composed of peels of orange, sweet lemon, banana, mango, pea pods and stubs of cauliflower/cabbage) obtained from ‘mandis’, juice shops and various cafeterias, was utilized for obtaining culture medium for *R. toruloides.* The collected waste material was shredded; 30 g of it was steamed in 100 mL water for 15 min to prepare an ‘extract’ that would be used as fermentation medium, hence called waste extract (WE) (Fig. 1). Seasonal variations in sugar content in WE was observed and so while preparing it each time, the C/N ratio was maintained at 30:1 to minimize this variation and also methods related to WE preparation and pretreatment were standardized and have been reported previously [27]. The various types of ‘mandi’ waste (orange/pea/mango/papaya peels; cauliflower/cabbage stubs) were analyzed individually for carbon (C) and nitrogen (N) content (data not shown). Peels with higher C content were added with those having appropriate N content to maintain the standardized C/N ratio of 30:1 in the resultant WE. C was determined by phenol sulphuric acid test [28], DNS for reducing sugar [29] and N by Kjeldahl method. After autoclaving, nutritional analysis of dried waste was done at Central Laboratory for Soil and Plant Analysis, Division of Soil Science and Agricultural Chemistry, ICAR-Indian Agricultural Research Institute, New Delhi (India).

### Cultivation of *R. toruloides*

A 48 h old seed culture (log phase) having optimal density (OD_600_) in the range of 0.6–0.8 was used to inoculate a 50 mL WE. Samples were collected at every 6–12 h interval for determination of OD (600 nm), dry and wet cell weight, total sugar [28] and reducing sugar [29] of the medium; lipid content and β-carotene content of the biomass. The cells were harvested by centrifugation using pre-weighed tubes, at 4000 rpm for 10 min; supernatant was discarded and harvested cells were weighed (wet cell weight) and then dried in oven at 80 °C for 1–2 h, till dry and final weight of centrifuge tubes containing cells were obtained (~ 20% of wet weight). This is the dry cell weight (DCW) and is expressed in (g/L). DCW = (W_2_ − W_1_)/V_1_; where: W_2_ = weight of tube containing dried cells, W_1_ = weight of empty tube and V_1_ = Volume of culture [30, 31]. Biomass was assessed by absorbance (OD 600 nm) and DCW of pellet. These parameters were further used to calculate kinetic parameters such as t_d_ (doubling time), t_g_ (generation time) and µ (specific growth).

### Analytical methods

#### Determination of lipid content

Extraction of lipid was done following the protocol of Bligh and Dyer [32]. The cells (100–1000 mg) were harvested by centrifugation at 10,000 rpm for 10 min at 4 °C, washed once with distilled water and the dry pellet weight was recorded (W). The dry pellet was resuspended in 3.75 ml of chloroform/methanol (2:1, v/v solution) and vortexed for 15 min, followed by 1.25 ml of chloroform and 1.25 ml of 1 M NaCl, with vortexing at every step.

The mixture was centrifuged at 3000 rpm for 15 min to separate the aqueous and organic phase. The organic lower phase was transferred to a weighed vial and dried till organic phase evaporated. The weight of the vial was again recorded (W2).

Therefore, lipid content from dry cell biomass was calculated as:

$${\text{Lipid content }}\left( {{\text{g}}/{\text{L}}} \right) \, = \frac{{{\text{Lipid weight in weighed vial }}\left( {\text{g}} \right) \, {-}{\text{ empty weighed vial }}\left( {\text{g}} \right)}}{{{\text{Grown in sample Volume }}\left( {\text{L}} \right)}}$$

$${\text{Lipid content }}\left( \% \right) \, = \frac{{{\text{Lipid content }}\left( {{\text{g}}/{\text{L}}} \right)}}{{{\text{Dry cell weight }}\left( {{\text{g}}/{\text{L}}} \right)}} \times { 1}00$$

$${\text{Lipid Productivity }} = \frac{{{\text{lipid concentration}}({\text{g}}/{\text{L}})}}{{{\text{Fermentation time }}\left( {\text{hrs}} \right)}}[{\text{unit}} - {\text{g}}/{\text{L}}/{\text{h}}]$$

#### Transesterification and FAME analysis

Transesterification of 0.15 g lipid (extracted from pelleted cells) was performed in a glass tube using 2 mL *n*-Hexane and 1 mL 2 M methanolic-KOH. The tubes were capped and vigorously shaken for 30 s and then incubated for 20 min at 70 °C. After cooling to room temperature 1.2 ml of 1 M HCl was added with gentle stirring and 1 ml of *n*-hexane was added and the mixture was allowed to stand for separation of phases. The upper yellow phase containing the FAMEs was transferred into the glass vial for further identification using gas chromatography (GC).

#### Gas chromatography

Gas chromatography was done to quantify and identify individual components present in transesterified samples. It was performed on a Rastech gas chromatography model 2806 (Delhi, India) equipped with 30 m long (film thickness 0.25 um; internal diameter 0.25 mm) GC column EC™^−1^ (Grace Davison Discovery Sciences) containing dimethyl polysiloxane as the stationary phase and a flame ionization detector with Winchrom (IndTech Instruments) acquisition software, under the following conditions: carrier gas N_2_ flow 1 mL/min, column pressure 2.05 bar, initial oven temperature was maintained at 200 °C for 5 min and then elevated to 280 °C at 10 C min^−1^, injector temperature 250 °C, detector temperature 275 °C with 2 kg/cc air flow. Identification and quantification of methyl esters were based on the comparison of retention times and peak areas of commercial standards, sample volume used was 0.2 µL (diluted with hexane).

#### GC-mass spectrographic analysis

Methyl esters of fatty acids were analyzed by a Gas Chromatography–Mass spectrometry on a Shimadzu GCMS—QP 2010 Plus (Shimadzu, Japan) fitted with a SP–2560 capillary column (100 m × 0.25 mm i.d). The temperatures of injection and detector ports were set at 260 °C. The oven temperature programmed was initially at 140 °C for 5 min, then rose at 4 °C/minute to 240 °C and finally held at 240 °C for 5 min. The carrier gas was nitrogen with a total flow rate of 16.3 mL/min. MS conditions: Ionization voltage was 70 eV; ion source temperature was 270 °C and mass range was 30–700 mass units. The individual peaks were identified by comparing their retention indices with standard chromatogram and their mass spectra with NIST/Wiley library of mass spectral database.

#### Carotenoid extraction

Carotenoids were extracted using a modified method of Kim and Park [33]. Cells were collected by centrifugation, washed and treated with DMSO, incubated at 50 °C for 1 h and the process was repeated till all the carotenoids were extracted in DMSO. The carotenoids were then extracted in the hexane phase by liquid–liquid extraction. The OD of the supernatant was determined at 450 nm against hexane using a Systronics UV–Vis spectrophotometer 117. Quantification was done using standard curve of β-carotene (prepared in hexane). β-carotene was quantified as it is present as one of the components of yeast carotenoids. All statistical calculations were done using *MEDCALC Software bvba* statistical software (www.medcalc.org).

#### HPLC analysis

For detection of different carbon sources such as glucose, xylose, glycerol, arabinose etc. in the WE, HPLC was performed using 5 mM H_2_SO_4_ in Milliq water as mobile phase in a HPLC column AMINEX HPX-87H, 300X7.8MM that is specially designed for ethanol and organic acids estimation. The flow rate was maintained at 0.5 mL/min. HPLC analysis was used to determine the presence of various sugars in the WE and changes in their concentration after fermentation.

## Results

### Cell Growth and glucose utilization of *R. toruloides* grown in ‘extracts’ made from waste(s)

**Fig. 2:**
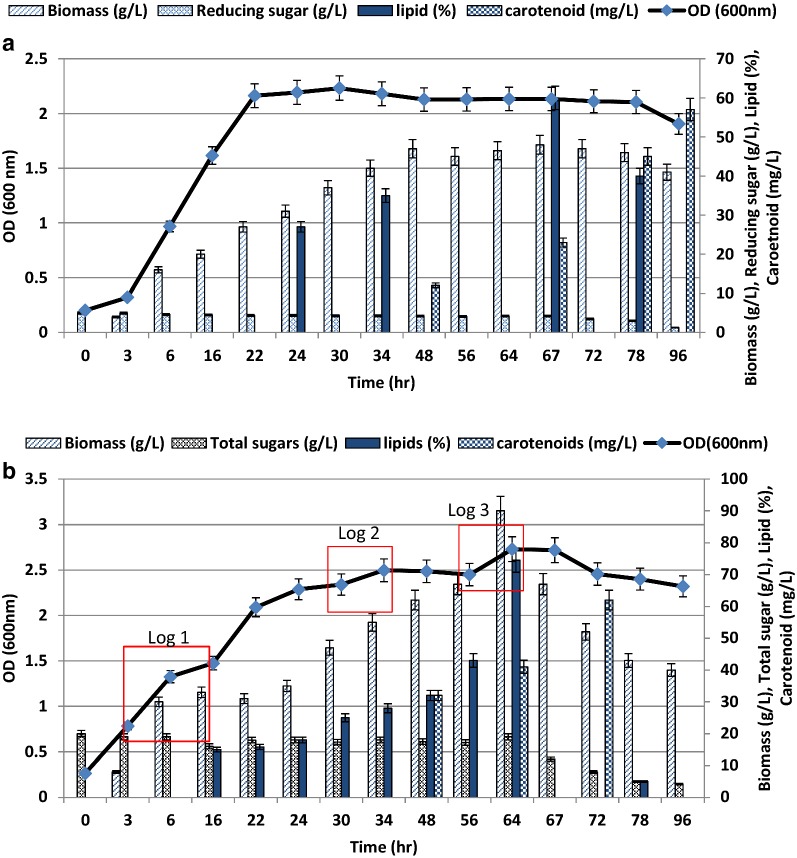
Comparative study of growth, lipid and carotenoid production during batch cultivation of *R. toruloides* in **a** Minimal medium **b** Waste ‘extract’

Nutritional analysis of the waste ‘extract’ (WE) showed the presence of 30% carbon, 1% nitrogen, 0.18% phosphorus and 0.56% potassium. *R.toruloides* showed a triauxic growth pattern when grown on WE but not when grown in minimal medium (MM) containing glucose as the only carbon source (Fig. 2a, b). The total sugar content of WE at the beginning of the experiment (0 h reading) was ~ 20 g/L (Fig. 2b), comprising of 48.55% glucose, 30.9% xylose and 20.4% glycerol apart from smaller amounts of arabinose, xylitol, etc. as determined by HPLC analyses. Various agro-industrial residues, such as monosodium glutamate [34], methanol [35], molasses/crude glycerol [36], soluble starch [37] have been reported as growth media for producing lipid by various oleaginous microorganisms (Table 1). All of these waste sources showed the presence of a single carbon source; however, WE contained multiple carbon sources, such as arabinose, xylohexose, xylitol, succinate acid etc. with glucose, xylose and glycerol as predominant components. *R. toruloides* efficiently utilized more than one carbon source for growth and lipid formation as also indicated by its growth curve.

**Table 1:** Comparative study of lipid production from different strains of oleaginous yeast

Oleaginous yeast strain	Carbon source	Growth medium	Biomass (g/l)	Lipid content (% dcw)	References
*Rhodospordium toruloides*	Glucose/xylose/glycerol	Waste ‘extract’	18	74.5	The present work
*Rhodotorula glutinis*	Glucose	Monosodium glutamate (MSG) wastewater	25	20	[34]
*Candida lipolytica*	Methanol	NA^a^	NA^a^	4.9	[35]
*Yarrowia lipolytica*	Molasses and crude glycerol	YPD	50 cdw	31	[36]
*Cryptococcus terricola* JCM 24523	Soluble starch	YPD	4.88	61.96	[37]
*Rhodosporidium toruloides* AS2.1389	Wastewater	YPD	8.12 ± 0.23	43.65 ± 1.74	[30]
*Rhodotorula glutinis* TR29	Molasses	Malt extract broth (MEB)	16.2	64.8	[46]
*Yarrowia. lipolytica* B9	Deproteinized whey	DWB medium	7.4	58	[47]
*Metschnikowia pulcherrima*	Glycerol	OMP medium	7.4	40	[48]
*Cryptococcus curvatus* ATCC 20509	Corn stover	YPD liquid medium	27.7	43.6	[49]

^a^ Comparative results were analyzed according to dry cell (W/V)

Analysis of the growth curve of *R. toruloides* using WE as culture medium showed that there was a significant increase in cell mass after 6 h of cultivation (Fig. 2b), whereas in case of MM, the increase was observed after 22 h. The log phase progressed from 6 h to 16 h, followed by a short stationary phase after 24 h and then the second log phase progressed from 30 h to 48 h, followed by a third log phase (~ 56 h to 72 h). Different growth phases log 1, log 2 and log 3 supported growths at the rate of 0.109/h, 0.043/h and 0.039/h (Table 2). HPLC analysis (Additional file 1: Table S1A) and (Additional file 1: Figure S1A) of the cell-free fermentation medium was performed at various time points during growth, to evaluate the utilization of glucose, xylose, glycerol, etc. Results showed that ~ 80% glucose was consumed (10.9 g/L to 2.6 g/L in 24 h) during the initial log phase and preferably used as main carbon source along with slight co-consumption of xylose and glycerol (~ 5% of each in 24 h). HPLC data and kinetic growth rate variations supports the ‘triauxic’ mode of growth where the organism preferentially used a particular sugar faster than the others, during the three log phases in WE (Table 2). We observed that during log phase 3, biomass production was enhanced and the productivity was the highest (0.20 g/L/h) (Table 2). Glycerol and other carbon sources were probably used during the 3^rd^ log phase, as they were rapidly consumed toward the end (72 to 80 h). Glucose might have been valuable as a growth initiator but higher productivities were observed during the third log phase, which may be attributed to higher solubility and slower catabolism of glycerol. Between glucose and xylose a diauxic pattern of growth was reported earlier [38] in which one substrate was catabolized preferentially over the other with a lag phase occurring between the growth phases.

**Table 2:** Kinetic parameters of *R. toruloides* during growth in minimal medium and waste extract

Growth Medium	Growth phase	Specific growth rate (µ) (h^−1^)	Doubling time (t_d_) (h)	Yield (Y_x/s_)	Yield (Y_p/x_)	Yield (Y_p/s_)	Productivity (g L^−1^ h^−1^)
1. Minimal medium	Log	0.093	7.44	0.91	0.6	1.37	0.110
2. Waste ‘extract’	Log 1	0.109	6.35	0.41	0.15	0.06	0.06
	Log 2	0.039	17.63	0.61	0.28	0.17	0.09
	Log 3	0.043	15.87	0.94	0.74	0.70	0.20

After growing for about 72 h, there was a constant decrease in OD until 96 h, suggesting decrease in cell viability and growth. Yield_(X/S_) was highest during log phase 3 (0.94 g/g) as compared to log phase 1 (0.41 g/g), followed by log phase 2 (0.61 g/g) as it is the preferred carbon source for this strain (Table 2).

### Lipid production by *R. toruloides* grown in extracts made from ‘mandi’ waste

When grown on WE, initially the lipid content increased significantly, as indicated by lipid saturated cells. Figure 2b shows the lipid production profile of *R. toruloides* at different time intervals. Lipid content calculated initially at 22 h was 4.898 g/L i.e. 15.8% of DCW and highest lipid content was 13.41 ± 2.50 g/L i.e. 74.5% of 18 g/L DCW. Highest lipid content determined in case of MM was 5.76 ± 0.78 g/L i.e. 60% of 9.6 g/L DCW. The significance level (p value) calculated form *t* test was 0.003.

The consumption of carbon source(s), cell growth and lipid production demonstrated adequate utilization of all available growth substrates by *R. toruloides.* Productivity of lipids in WE during triauxic growth was calculated as 0.20 g/L/h in duration of 67 h (as described in the methods). GC and GC–MS analysis confirmed the occurrence of long chain fatty acids namely myristic, palmitic, palmitoleic, oleic, stearic and linoleic acid present in various amounts (Tables 3 and 4). The content of oleic acid, lauric acid, stearic and linoleic acid varied when cultivated in MM and WE. As determined by GC–MS, cells cultivated in WE showed 44.63% increase in oleic acid and 13.39% in linoleic acid respectively, as compared to cells grown in MM. Stearic, linoleic acid and oleic acid and their uses for making soaps, cosmetics, agricultural chemicals, etc. are depicted in Table 4. The presence of such important products may be made cost effective by standardizing its production on inexpensive nutrient sources such as WE.

**Table 3:** The fatty acids content in the lipid of *R. toruloides* obtained from GC analysis

Lipid composition	Fatty acid type	Content of fatty acid (%)
Lauric acid (C12)	Saturated fatty acid	2.02
Myristic acid (C14)	Saturated fatty acid	4.23
Palmitic acid (C16)	Saturated fatty acid	13.42
Margaric acid (C17)	Saturated fatty acid	2.83
Oleic acid (C18:1)	Mono-unsaturated fatty acid	32.6
Linoleic acid (C18:2)	Poly-unsaturated fatty acid	22.95
Others		21.95

**Table 4:** Fatty acid composition (FAMES) as determined by GC–MS analysis

Compound name & symbol	Fatty acid type	Waste ‘Extract’	Minimal media	Uses
C_12.0_ (Lauric acid/dodecanoic acid)	Saturated fatty acid	2.02	3.03	Long shelf-life, non-toxic, treatment for acne, production of soaps and cosmetics [50]
C_14.0_ (Myristic acid/ tetradecanoic acid)	Saturated fatty acid	4.23	4.49	Myristic acid acts as a lipid anchor in biomembranes. The ester isopropyl myristate is used in cosmetic and topical medicinal preparations [51]
C_16.0_ (Palmitic acid/hexadecanoic acid)	Saturated fatty acid	13.42	16.27	Used in production of soaps, cosmetics, release agents, processed food, natural additive in organic products. Papiperidone palmitate used in the treatment of schizophrenia [49, 52]
C_16.1_ ω9c (Palmitoleic acid/9*Z*)-hexadec-9-enoic acid)	Omega 7-monounsaturated fatty acid	12.1	13.79	Adhesives and sealant chemicals, Agricultural chemicals (non-pesticidal), Finishing agents, Lubricants and lubricant additives, Surface active agents. Also used as supplements and vitamins [50, 53]
C_17.0_ (Margaric acid/Heptadecanoic acid)	Saturated fatty acid	2.83	5.50	Adhesives and sealant chemicals, Finishing agents, lubricants, agricultural chemicals (non-pesticidal), surface active agents, flllers, and solvents
C_17.1_ ω8c (cis-10-Heptadecenoic acid methyl ester)		0.64	3.67	–
C_18.0_ (Stearic acid/octadecanoic acid)	Saturated fatty acid	9.21	10.47	Soaps, cosmetics, detergents, lubricants, softening and release agents. Plaster castings, manufacture of lead-acid batteries, candle-making, used along with simple sugar or corn syrup as a hardener in candies, dietary supplements, fireworks [54]
C_18.1_ω9c (Oleic acid/cis-9-Octadecenoic acid)	Monounsaturated omega-9 fatty acid	32.60	22.54	Soap as an emulsifying agent, emollient, excipient in pharmaceuticals, and as an emulsifying or solubilizing agent in aerosol products, soldering flux in stained glass work for joining lead came [55, 56]
C_18.2_ (Linoleic acid/9, 12 Octadecadienoic acid)	Polyunsaturated omega-6 fatty acid	22.95	20.24	Used in making quick-drying oils, useful in oil paints and varnishes, beauty products industry. Anti-inflammatory, acne reductive, and moisture retentive properties [57–60]

### β-Carotene production by *R. toruloides* grown in extracts made from ‘mandi’ waste

In the early growth phase, no carotenoid production was observed in either the WE or MM because it is a secondary metabolite. After 45 h of cultivation, carotenoid production started as the culture reached its stationary phase of growth. Maximum carotenoid production was observed after 72 h and 100 h of growth in WE and MM respectively. β-carotene production was determined as 62 ± 1.70 mg/L when cultivated on WE as compared to 57 ± 2.18 mg/L on MM. A significance value of 0.0251 (P-value), and 95% Confidence Interval (CI) of the difference was established (MEDCALC Software bvba). Earlier reports showed high carotenoid production (1.10 mg/L β-carotene) from *Rhodotorula glutinis* DBVPG 3853 [39], and lower production in brewery waste waters ranging between 0.6 and 1.2 mg/L of total average carotenoid content (∼ 50% β-carotene) [40]. In this study *R. toruloides* cultivated in WE showed an intermediate production (62 mg/L) when compared with earlier reports.

## Discussion

*Rhodosporidium toruloides* is an important oleaginous yeast known for higher lipid production and value added chemicals as it can grow on glucose, xylose and other sugars found in the hydrolysate of ‘waste’ biomass including lignocelluloses [41, 42].

Various oleaginous yeast have been cultivated in other inexpensive media to produce lipids, such as *Yarrowia lipolytica* was grown in molasses (waste generated by sugarcane industry), oleaginous filamentous fungus *Aspergillus oryzae* in potato processing wastewater (starch as carbon source), *Cunninghamella echinulata* on tomato waste hydrolysate (TWH) media (glucose as carbon) [36, 43, 44]. In all these cases there is primarily one carbon source (Table 1). WE used in this study contained mixed sugars; glucose, xylose, glycerol etc. which were sequentially utilized by *R. toruloides* to synthesize lipids during triauxic growth. The WE medium is inexpensive as compared to synthetic medium and richer in nutrients as compared to other waste resources (stover, molasses, etc.) and appropriate in terms of techno-economic utility of waste to bio-product.

*Rhodosporidium toruloides* has the potential to produce lipids from ‘mandi’ waste prepared by steaming inedible parts of fruits and vegetables that otherwise pose serious environmental problem. Various sugars in the hydrolysate (depolymerized by steaming) consisted of glucose and xylose that were sequentially metabolized in order of preference by *R. toruloides.* This pattern of growth was reported earlier in *R. minuta* and in *R. glutinis* where diauxic growth is suggested respectively, with ~ 40% and 36.4%  % lipid accumulation [38]. However, earlier study showed that *T. cutaneum* accumulated 59% lipids by utilizing glucose and xylose simultaneously [45], suggesting that utilization of sugars is strain dependent and varies with C/N ratio and that sequential or simultaneous utilization of saccharides do not impact accumulation of lipids (Table 1). When cells are grown in a medium that is deficient in a key nutrient, usually nitrogen, they cease to proliferate due to the lack of biosynthesis of proteins and nucleic acids. The excess carbon substrate however, continues to be assimilated by the cells and converted into storage fat (TAGs, sterols, etc.). This phenomenon is common in filamentous fungi and oleaginous yeast strains. However, if the C/N ratio is higher than 80 g/g NH3+ , there is a prolonged nitrogen deficiency, thus altering lipid synthesis and directing the metabolic flux toward the production of products such as citric acid. Comparatively product yield (*Y*_P/S_) during batch fermentation at high substrate concentration showed that the increase of glucose concentration resulted in the decrease of product yield due to less cell growth. This phenomenon therefore posed some difficulty for the up scaling lipid production due to high substrate consumption rate in the presence of high C/N ratio (i.e. high concentration of glucose with lower level of nitrogen) due to reduction in biomass production. High cell density cultures via fed batch fermentation provide an alternative to achieve higher product yields.

For the production of biodiesel, saturated fatty acids and monounsaturated fatty acids are more valued over saturated fatty acids e.g., C16:0 and C18:0 which are solid at room temperature and can be difficult to use in cold weather for production of biodiesel. On the other hand, monounsaturated fatty acids have better flow properties as they are liquid at room temperature, and hence preferable for biodiesel (27). There are some more higher-value oleochemical products such as nutritional polyunsaturated fatty acids that have a higher profit margin and may be suggested as economically viable products derived from oleaginous yeasts, as compared to biodiesel and might soon become acceptable to industries dealing in solvents, adhesives, lubricants, or platform chemicals.

## Conclusion

‘Mandi’ waste is a rich amalgamation of various carbon source(s) utilized in culture medium. *R. toruloides* showed triauxic growth when cultivated in it and consequently accumulated higher amounts of lipids and carotenoids. Our results suggest that this culture medium can support in 24.17% increase in lipid and 8.77% increase in carotenoid production as compared to synthetic minimal medium.

The waste ‘extract’ also contains the essential mineral salts required for growth of the organism and therefore serves as a complete medium (supplemented fermentation medium) in it. This bioprocess is suitable for waste management, is environment friendly and gives good return upon investment (ROI).

## Additional file

**Additional file 1: Table S1.** HPLC analysis of fermentation medium at various time points. Retention time and area compared with standard area to determine concentrations of glucose, xylose and glycerol (g/L). **Figure S1.** HPLC analysis of waste ‘extract’ at different time points during the cultivation of *R. toruloides,* to determine the utilization of various carbon sources. Blue box indicates glucose; red indicates xylose and black indicates glycerol.

## Abbreviations

WE: waste extract
MM: minimal medium
HPLC: high performance liquid chromatography
OD: optimal density
DMSO: dimethyl sulpho-oxide
FAMES: fatty acid methyl esters
GC-MS: gas chromatography-mass spectrometer
