# Correction to: Triauxic growth of an oleaginous red yeast *Rhodosporidium toruloides* on waste ‘extract’ for enhanced and concomitant lipid and β‑carotene production

**DOI:** 10.1186/s12934-019-1082-4

**Published:** 2019-02-18

**Authors:** Gunjan Singh, Sweta Sinha, K. K. Bandyopadhyay, Mark Lawrence, Ram Prasad, Debarati Paul

**Affiliations:** 1 Amity Institute of Biotechnology, Amity University, Sec 125, Noida, Uttar Pradesh 201313 India; 2 Basic Sciences, College of Veterinary Medicine, Mississippi State University, Mississippi State, MS 39762 USA; 3 School of Environmental Science and Engineering, Sun Yat-Sen University, Guangzhou, 510006 China

## Correction to: Microb Cell Fact (2018)17:182 10.1186/s12934-018-1026-4

Upon publication of this article [1], the authors realized that one of the coauthor name was accedentally deleted during the review process and not presented in the original version of the article. The coauthor Dr. Ram Prasad should be listed as fifth author. The corrected author group is published with this erratum.

## References

[1] Singh G, Sinha S, Bandyopadhyay KK, Lawrence M, Prasad R, Paul D (2018). Triauxic growth of an oleaginous red yeast *Rhodosporidium toruloides* on waste ‘extract’ for enhanced and concomitant lipid and β-carotene production. Microb Cell Fact.

