# Supplementary material for: Triauxic growth of an oleaginous red yeast Rhodosporidium toruloides on waste ‘extract’ for enhanced and concomitant lipid and β-carotene production
Source: Microb Cell Fact. 2018 Nov 19;17:182. doi: 10.1186/s12934-018-1026-4 (PMC6240951; doi:10.1186/s12934-018-1026-4)
Supplement: Supplementary file 1 — Additional file 1: Table S1. HPLC analysis of fermentation medium at various time points. Retention time and area compared with standard area to determine concentrations of glucose, xylose and glycerol (g/L). Figure S1. HPLC analysis of waste ‘extract’ at different time points during the cultivation of R. toruloides, to determine the utilization of various carbon sources. Blue box indicates glucose; red indicates xylose and black indicates glycerol. [file 12934_2018_1026_MOESM1_ESM.docx]

**Additional file**

**Table S1**. HPLC analysis of fermentation medium at various time points. Retention time and area compared with standard area to determine concentrations of glucose, xylose and glycerol (g/L).

| Carbon Source | Glucose  (R.T: 18.0975) | | Xylose  (R.T: 19.429) | | Glycerol  (R.T: 27.10) | |
| --- | --- | --- | --- | --- | --- | --- |
| Standard area | **24708536 (a)** | | **28418932 (a)** | | **12019077 (a)** | |
| Sample (hr) | **AREA (b)** | **T.conc (g/L) (c)*** | **AREA (b)** | **T.conc (g/L) (c)*** | **AREA (b)** | **T.conc (g/L) (c)*** |
| 0 | 54101508 | 10.9 | 39573948 | 6.95 | 11057551 | 9.2 |
| 8 | 34236498 | 6.5 | 34818798 | 6.1 | 6850874 | 5.7 |
| 24 | 13015649 | 2.6 | 9215981 | 1.6 | 5648966 | 4.7 |
| 32 | 5387215 | 1.05 | 9215981 | 1.5 | 5408585 | 4.5 |
| 48 | 3748923 | 0.75 | 7395122 | 2.6 | 3485532 | 2.9 |
| 56 | 567555 | 0.1 | 7220619 | 2.5 | 2884578 | 2.4 |
| 72 | ND | 0 | 6752328 | 0.7 | 2163434 | 1.8 |
| 80 | ND | 0 | 1400090 | 0.4 | 2163434 | 1.8 |
| 96 | ND | 0 | 513787 | 0.1 | 2163434 | 1.8 |

*c = (b÷a)×5 [as the sample was diluted 5 times]

**Figure S1**. HPLC analysis of waste ‘extract’ at different time points during the cultivation of *R. toruloides,* to determine the utilization of various carbon sources. Blue box indicates glucose, red indicates xylose and black indicates glycerol.


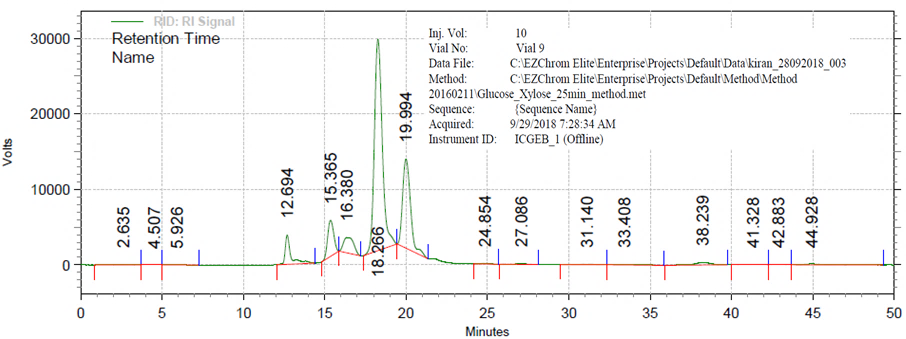


0h


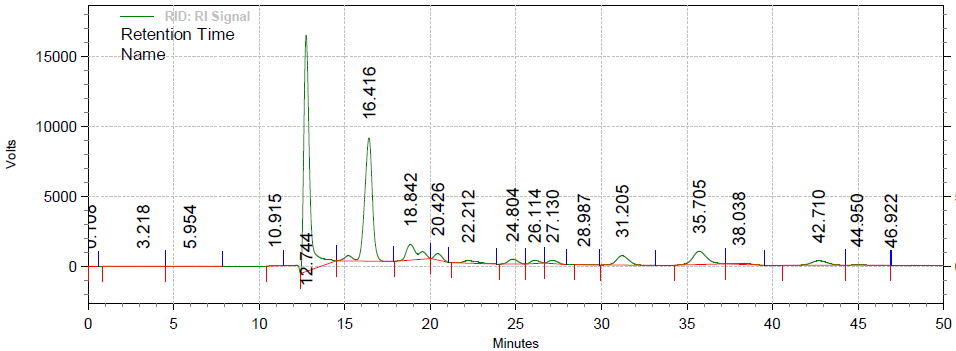


24h


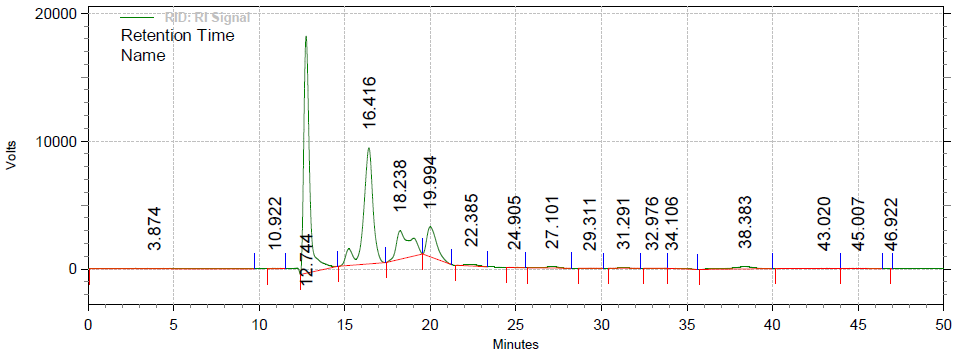


48h


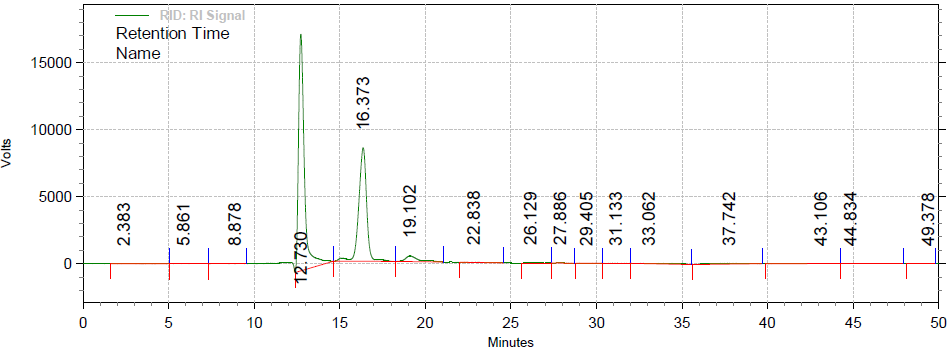


80h
